# Which is the best treatment on a 2 cm complete endophitic tumor on the posterior side of the left kidney?

**DOI:** 10.1590/S1677-5538.IBJU.2016.01.03

**Published:** 2016

**Authors:** Rodrigo Gobbo Garcia

**Affiliations:** Radiologista intervencionista, Centro de Imagem, Hospital Israelita Albert Einstein, São Paulo, Brasil

**Keywords:** Therapeutics, Neoplasms, Cryosurgery, Nephrectomy

## Opinion: Cryoablation

**The Clinical Problem**

With the increased use of advanced imaging techniques, incidental renal mass have become a very frequent finding (1).

Approximately 13 to 27% of abdominal imaging studies incidentally identify a renal lesion , a fact that makes suspected renal cell carcinoma be diagnosed at an early stage (2). Most excised small renal cancers are classified as low grade at the time of diagnosis and synchronous metastases are very infrequent finding associated to such small lesions (3).

Although partial nephrectomy remains the reference standard for treatment of small renal masses, the guidelines of the American Urological Association support consideration of thermal ablative techniques for the treatment of patients with T1a disease (< 4 cm) (4).

Furthermore, the development of ablative techniques has widened the range of treatment options available to these patients and international consensus panels support other indications for ablative therapy for renal tumors (patients with a increased risk of multiple RCC tumors – e.g. von Hippel–Lindau syndrome, clinical conditions not suitable for surgery and solitary or transplanted kidney) (5).

## TECHNICAL CONSIDERATIONS

Thermal ablation is performed by inserting needle applicators within the renal tumors to generate lethal temperatures to neoplastic tissues encompassed by ablation zone . Cryoablation and radiofrequency ablation are the most common methods (6).

**Figure 1:**
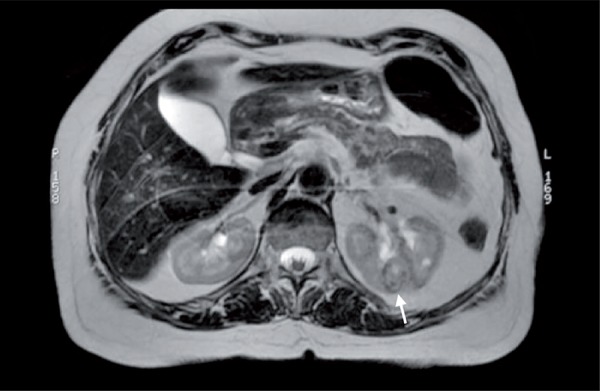
MR image, T2 sequence. Arrow: A biopsy-proven 2.2 cm clear cell carcinoma on the left kidney.

**Figure 2:**
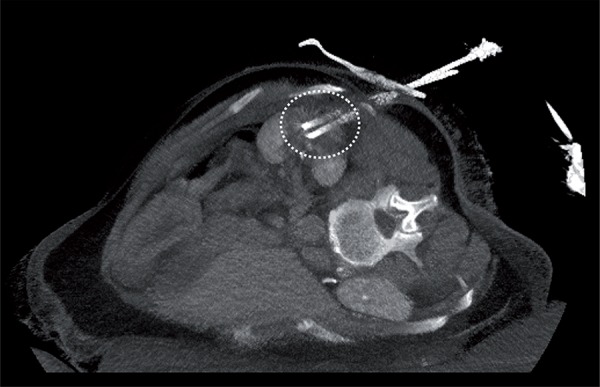
Interventional CT-scan. Cryoablation with two cryoprobes within target lesion. Dotted circle: edge of ice-ball.

**Figure 3:**
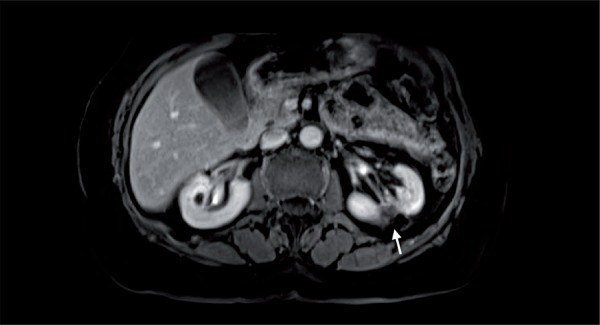
MR image, T1 post-contrast for surveillance two years post ablation. Arrow: Retracted and non-enhancing ablation zone, depicting successful treatment.

With refinements in probe size and design, a percutaneous image-guided approach may be preferable to a laparoscopic approach for thermal ablation, since procedure- associated morbidity would be lower (7) (Figures 1 - 3).

Historically, percutaneous ablation has been reserved for patients with small, exophytic tumors in the posterolateral kidney. However, the increased use of cryoablation and displacement techniques (8) (e.g. hydrodissection and pneumodissection - infusion of fluid or gas via a small-caliber catheter placed under image guidance ) have significantly expanded the number of renal tumors that can be successfully treated percutaneously, including larger tumors, central tumors, and tumors in less accessible locations within the kidney (9).

Cryoablation, rather than radiofrequency ablation, has shown significant promise in treating these larger more complex renal tumors (9).

As the name implies, cryoablation relies on low temperatures to induce cell death. The process of cryoablation obeys the Joule- Thomson effect whereby expanding of some gases (e.g. argon) within a needle-like chamber (the cryoprobre) produces heat sink near the antenna tip that cools the probe to temperatures of –160ºC or colder (10). The cell-lethal isotherm is between –20ºC and –40ºC. Slow freezing produces intra- cellular ice crystals, and fast freezing induces extracellular ice crystals. Both processes induce cell death by different cellular mechanisms. In addition, freeze-thaw cycles can induce cellular dehydration, membrane rupture, vascular thrombosis and tumor cell apoptosis (11).

Proximity of the tumor to the collecting system may represent a relative contraindication to cryoablation due to the risk of urothelial injury and ureteral strictures have been reported, particularly for tumors in the medial lower pole (10).

Placement of a ureteral stent with retrograde warm saline irrigation of the collecting system and a very reliable identification of ureter during the ice ball monitoring may mitigate this risk (12).

Freezing into calyceal structures or intrarenal pelvic collecting system did not cause any apparent strictures or vascular injuries in longer-term follow-up, similar to prior animal data (13). Relative warming of the ablation zone by large central vessels may limit the ability to achieve cytocidal temperatures at the central tumor margin, and more aggressive treatment with larger cryoprobes and a greater iceball margin is indicated (14).

Careful preprocedure cross-sectional imaging assessment of a candidate patient’s small renal mass is required to minimize complications and maximize therapeutic efficacy. A practical algorithm for procedure planning, ABLATE, has been proposed that takes into account the following tumor characteristics:

A, axial tumor diameter; B, bowel proximity; L, location within the kidney; A, adjacency to the ureter; T, touching renal sinus fat; and E, endophytic or exophytic position (15).

Of all tumor characteristics, the size of the renal mass is the most important factor in achieving local tumor control with ablation (16). This is primarily related to the small size of the ablative zones tissue generated by most ablation devices and some limitations in monitoring its size during the treatment. On that point of view, cryoablation is superior to RFA because the iceball is easily depicted by CT-scan, making more predictable volumes of treatment. The size and shape of the ice ball can be manipulated with multiple cryoprobes synergistically working (10).

Endophytic tumor position (tumor completely surrounded by renal parenchyma) can make ablation procedures more difficult and has been associated with increased local treatment failures. Gupta et al. (17) reported technical failure or recurrence during a mean 18-month follow-up for seven of 46 (15.2%) endophytic tumors versus five of 117 (4.3%) nonendophytic tumors treated by ablation (p=0.016). Small endophytic renal tumors that are not confidently visualized with intraprocedural unenhanced CT are particularly challenging to treat.

Ultrasound guidance, ultrasound-CT, or ultrasound-MRI fusion guidance or administration of IV contrast agent (iodinate for CT and microbubbles for ultrasound) may help with localizing endophytic tumors.

Concerning to the location of the renal tumors, an important potential complication to consider before ablation is nerve injury, which can lead to postablative neuralgia and paresthesias. In the context of renal ablation planning, one should consider the position of the intercostal nerves, genitofemoral nerve, and lateral femoral cutaneous nerves. The ablation of posterior masses located close to major psoas muscle draws attention to the danger of damaging the genitofemoral nerve, resulting in chronic pain, tenderness, and diminished sensitivity within the skin area of the ipsilateral groin (18). Displacement techniques (e.g. hydrodissection and torquing handle of the cryoprobe as a lever) can move away the tumor of the psoas, lowering the risks of neural injuries (19).

## RESULTS

Local control rates approaching 100% for tumors smaller than 3 cm in select series, with very low tumor recurrence rates (20).

Thompson et al recently compared the oncologic outcomes for cT1a tumors between RFA (n=180), PCA (n=187), and PN (n=1057). On univariable analysis, there was no difference in estimated 3 years disease-free survival between the three treatment modalities (98% for all three arms). Distant metastasis-free survival at 3 years, however, was better for PN (99%) and CA (100%) compared with RFA (93%) (21).

Whitson et al. (22) compared 7704 nephron-sparing surgeries and 1114 renal ablations using data from the Surveillance, Epidemiology, and End Results cancer registry and showed very similar 5 years disease-specific survival rates (98.3% vs 96.6%).

Given that long-term studies have shown durable outcomes for nephron-sparing surgery, the American Urological Association guidelines assert that the most important disadvantage of any ablative technique is a higher local recurrence rate relative to that of partial nephrectomy (4). However, this difference may be overstated in certain patient cohorts and is not entirely evidence based but rather due to a lack of long-term disease-free survival data on ablative management of small RCCs. Results of short- to medium-term studies suggest that appropriate patient selection can yield oncologic outcomes comparable to those of partial nephrectomy with the added benefit of improved preservation of renal function (5). Therefore, the decision to pursue ablative treatments rather than surgery requires careful consideration. Ideally, the advantages and risks of each approach must be compared and contrasted in consultation with the patient and the local tumor board.

Furthermore, the possibility of repeated ablations in residual lesions or *de novo* tumors, becomes even more atractive the percutaneous approach in comparison to surgery.

In addition, nephron-sparing surgery, including open partial nephrectomy and laparoscopic partial nephrectomy, can be more technically challenging and result in serious complications, such as excessive blood loss and urinary fistula formation (23).

The other obvious advantages of ablation relative to extirpative surgery include reduced morbidity; better preservation of renal parenchymal volume, which correlates with overall renal function; faster recovery time; shorter or no inpatient hospitalization; and possibly being the only treatment option available to patients with serious comorbid conditions who are not considered surgical candidates.

The cost to the health care system may also be reduced with increased use of noninvasive ablative techniques (24).

## CONCLUSION

Although nephrectomy (partial or radical) remains the reference standard for renal tumor treatment, physician acceptance and patient interest in percutaneous ablation are growing as intermediate and long-term outcome data become available.

Percutaneous cryoablation of renal tumors under imaging guidance is a curative alternative technique with a low complication rate. The best indications are tumor smaller than 4 cm, even though larger tumors can be effectively treated in selected cases. Percutaneous cryoablation is effective for preserving renal function, even in patients with single kidney. Moreover, in cases of incomplete treatment, cryoablation can be repeated and does not preclude salvage surgery.

So why not cryoablation as a first line therapy for a 2 cm complete endophitic tumor on the posterior side of the left kidney?
